# Prognostic value of liver stiffness in patients hospitalized for acute decompensated heart failure: a meta-analysis

**DOI:** 10.1007/s40477-024-00873-0

**Published:** 2024-03-18

**Authors:** Noemi Macerola, Laura Riccardi, Enrico Di Stasio, Massimo Montalto, Antonio Gasbarrini, Maurizio Pompili, Matteo Garcovich

**Affiliations:** 1 Division of Internal Medicine, San Carlo di Nancy Hospital, GVM Care and Research, Rome, Italy; 2 Diagnostic and Interventional Ultrasound Unit, CEMAD Centro Malattie dell’Apparato Digerente, Medicina Interna e Gastroenterologia, Fondazione Policlinico Universitario A. Gemelli IRCCS, Rome, Italy; 3 UOC Chimica, Biochimica e Biologia Molecolare, Fondazione Policlinico Universitario A. Gemelli IRCCS, Rome, Italy; 4 Dipartimento di Scienze biotecnologiche di base, cliniche intensivologiche e perioperatorie, Università Cattolica del Sacro Cuore, 00168 Rome, Italy; 5 Dipartimento di Medicina e Chirurgia Traslazionale, Università Cattolica del Sacro Cuore, 00168 Rome, Italy

**Keywords:** Shear wave, Elastography, Heart failure, Fibrosis, Cirrhosis, Liver stiffness

## Abstract

**Purpose:**

Heart failure (HF) is a major health problem affecting millions of people worldwide. In the latest years, many efforts have been made to identify predictors of poor prognosis in these patients. The aim of this systematic review and meta-analysis was to enlighten the correlation between liver stiffness (LS), assessed by Shear Wave Elastography techniques, and HF, particularly focusing on the prognostic value of LS on cardiovascular outcomes.

**Methods:**

We searched the PUBMED databases (up to May 1st, 2023) for studies that enlightened the correlation between LS and cardiovascular outcomes in patients hospitalized for acute decompensated heart failure (ADHF). We performed a meta-analysis to estimate the efficacy of LS in predicting the prognosis of patients with ADHF.

**Results:**

We analyzed data from 7 studies, comprising 677 patients, that assessed the prognostic value of LS in predicting cardiovascular outcomes in patients hospitalized for ADHF. The pooled analysis showed that increased liver stiffness was associated with higher risk of adverse cardiac events (hazard ratio 1.07 [1.03, 1.12], 95% CI).

**Conclusion:**

Increased LS is associated with poor prognosis in patients hospitalized for HF and might help effectively identify those patients at high risk for worse outcomes.

## Introduction

Heart failure is a major health problem affecting millions of people worldwide, with an increasing incidence in elderly patients and strong impact in terms of morbidity, mortality and cost [1]; it accounts for 5% of all hospital admissions, and 30–45% of patients hospitalized with acute decompensated heart failure (ADHF) die within 1 year [2].

Despite the efforts to prevent and modify the course of the disease, results are still far from being satisfying and there is a lack of parameters with strong prognostic power in terms of re-hospitalization and mortality for cardiovascular causes.

Regardless of the underlying left ventricle dysfunction degree [3], from 20 to 60% of HF patients develop right ventricle (RV) failure [4], with a volume and pressure overload which is transmitted to the venous system and increase right-sided filling pressures [5]. Venous congestion is both a prognostic indicator and an integral player in the pathogenesis of HF and of other organ dysfunction, since it contributes to worsening liver function [6, 7].

In fact, HF and liver diseases commonly occur together [8]. Hepatic damages secondary to heart failure may be seen both as “congestive hepatopathy”, due to elevated right-sided filling pressures [9], and ischaemia due to left ventricular failure (ischaemia–reperfusion injury) [10]. Frequently, these damages can occur together and, when perpetuated, they can lead to a chronic overlap culminating in liver fibrosis and cirrhosis [11]. The establishment of liver dysfunction worsens outcome in HF [12].

Right heart catheterization is the gold standard for measuring central venous pressure (CVP) [13]; however, it is an invasive procedure, not always available and not suitable to follow up patients [14]. An indirect, non-invasive and reliable parameter of right atrial pressure may be found in liver stiffness measurement (LSM) with shear wave elastography (SWE) ultra-sonographic techniques [15].

SWE is capable to provide “biochemical” information concerning tissue quality, and quantitative SWE is a non-invasive tool useful to diagnose the degree of liver fibrosis in chronic liver diseases [16]. In the liver, SWE has been shown to reflect increased right-sided filling pressure in HF patients without comorbid liver diseases [17], suggesting that liver stiffness (LS) assessed by SWE is a useful index for intrahepatic congestion and systemic volume status in HF [18].

SWE includes transient elastography (TE) and acoustic radiation force impulse (ARFI) techniques. With TE, the stress that generates the shear wave is applied on the body surface, and the measurement is performed in a fixed region of interest (ROI); this system does not display a B-mode image. With the ARFI based techniques, the shear waves are generated by the push-pulse of an ultrasound beam directly focused into the body. The B-mode image provides anatomical information, used to identify the best area for LS measurement. The measurement is performed in a small fixed ROI without an elasticity image, as in point SWE (pSWE), or in a larger ROI in which the elasticity values are color-coded, as in two-dimensional SWE (2D-SWE) [19].

Elevated LSMs are associated with hepatic congestion secondary to increased CVP and right-sided filling pressures of the heart, which are both known to be prognostic indicators in HF [14].

To date, still little is known about the clinical significance and the prognostic value of SWE in patients with HF. Some small observational studies have provided an overview about the possible role of SWE in the setting of ADHF and chronic congestive HF. The aim of this systematic review and meta-analysis is to evaluate the association of LS and cardiovascular outcomes in patients hospitalized for ADHF.

## Methods

This systematic review and meta-analysis were performed in accordance to the Preferred Reporting Items for Systematic Reviews and Meta-Analyses (PRISMA) statement [20]. The PICOS strategy was used, which comprised the following:

Population: patients hospitalized for HF;

Intervention (or exposure): LSM assessed by a SWE technique;

Comparison: not applicable;

Outcome (primary): re-hospitalization/morbidity/mortality;

Study type: Peer-reviewed observational, cohort and case–control studies.

There was no funding agency for this study. This systematic review and meta-analysis did not require ethical approval/informed consent, as it aggregated data from published literature, and there was no direct contact with individual patients.

### Search strategy and eligibility criteria

The search strategy comprised a combination of the following keywords (liver stiffness OR liver elastography OR liver elasticity) and (acute decompensated heart failure) and (outcome OR prognosis). For reliability, two reviewers (MG and NM) independently analyzed the currently available literature through systematic and comprehensive PubMed databases searching up to 01 May 2023. Any disagreement about study eligibility was resolved by discussion with a third review author (LR) until consensus. Reviews, in vitro studies, animal studies, autoptic studies and conference abstracts were excluded. The reference lists of the included articles were hand-searched to identify additional studies of interest. Full texts of all the potentially eligible studies were obtained by the two reviewers and evaluated for inclusion.

We included studies that reported the outcome of hospitalized patients with established HF (without prior history of chronic liver diseases) who underwent LSM during the hospital stay.

### Outcome

The primary outcome was the occurrence of one of the following: readmission for HF, death, worsening HF or a composite outcome (death/heart transplantation/Left Ventricular Assist Device).

### Study quality assessment

To assess the risk of bias, two authors (NM and MG) independently used the Newcastle–Ottawa Scale for comparative nonrandomized studies corresponding to each study’s design (cohort/cross-sectional) [21]. Such scale is a validated quality assessment instrument for non-randomized trials which evaluates three parameters of study quality: selection, comparability and exposure assessment. The scale assigns a maximum score of 4 for selection, 2 for comparability, and 3 for exposure, for a maximum total score of 9. Studies with a total score of ≥ 5 or ≥ 7 were considered to be of moderate or high quality, whereas those with a score of less than 5 were considered low-quality studies with high risk of bias. The scale results were tabulated in Table 1.

**Table 1:** Risk of bias assessment (Newcastle–Ottawa scale for non-randomized studies)

Study	Selection	Comparability	Outcome	Total score
Saito (2018)	3	2	3	8
Omote (2019)	3	2	3	8
Soloveva (2019)	3	2	3	8
Taniguchi (2019)	3	2	3	8
Panchani (2021)	3	1	3	7
Saito (2021)	3	2	3	8
Wang (2022)	3	2	3	8

### Statistical analysis

The statistical analysis was performed by using Review Manager (Version 5.5; Cochrane Collaboration, Oxford, UK) and Microsoft Excel (Version 16.45). Because of high heterogeneity, hazard ratios and their 95% confidence intervals (CIs) from individual studies were converted to log hazard ratios and corresponding standard errors, which were then pooled together using a generic invariance weighted random effects model. Statistical heterogeneity among studies was assessed with Cochran’s *Q* and quantified with Higgins *I*^2^ statistic [22, 23]. We considered an *I*^2^ of < 25% as low heterogeneity, *I*^2^ of 25–75% as moderate heterogeneity and *I*^2^ > 75% as high heterogeneity. Publication bias was assessed graphically using funnel plots.

## Results

The searches identified 34 potentially relevant papers, 20 after duplicates were removed. After title and abstract screening, only 11 full-text studies were considered potentially eligible for inclusion and 4 studies were then excluded for the following reasons: non hospitalized patients (*n* = 1); use of shear wave dispersion rather than LS (*n* = 3).

According to the Newcastle–Ottawa Scale assessing the risk of bias, all the included studies were of moderate-high quality (Table 1).

Seven studies met criteria and were included in our meta-analysis. The characteristics and most relevant findings of the included studies are summarized in Table 2.

**Table 2:** Baseline characteristics of included studies

Author (year), country	*N*	Study design	Method for LS assessment	Follow-up in days (IQR)	Variables adjusted for in the primary outcome	Primary outcome				**Median LS value**
						Readmission for HF	Death	Worsening HF	Death/HT/LVAD	
Saito (2018), Japan	105	P, SC	TE	153 (83–231)	Age, sex, NT-proBNP	31	11	na	na	8.8 kPa
Omote (2019)	70	P, SC	VTQ	272 (122–578)	Atrial fibrillation, NYHA class III or IV	na	5	21	na	1.48 m/s(no event); 2.24 m/s (event)^a^
Soloveva (2019)	149	P, SC	TE	289 ± 108^b^	Unadjusted	42	28	na	1	12.2 kPa
Taniguchi (2019)	171	P, SC	TE	203 (67–429)	Unadjusted	33	8	na	na	5.6 kPa
Panchani (2021)	52 (49)	P, SC	2D-SWE	365	Unadjusted	na	na	na	21	15 kPa
Saito (2021)	80	P, SC	2D-SWE	212 (82–275)	Unadjusted	25	3 (after readmission)	na	na	8.5 kPa
Wang (2022)	53	P, SC	TE	730 (149–730)	Unadjusted	24	3 (after readmission)	na	na	6.9 kPa^a^

*N* number of patients, *LS* liver stiffness, *IQR* interquartile range, *HF* heart failure, *HT* heart transplantation, *LVAD* left ventricular assist device, *P* prospective, *SC* single centre, *TE* transient elastography (fibroscan), *na* not applicable, *VTQ* virtual touch quantification (point-SWE), *2D-SWE* two-dimensional shear wave elastography

^a^Arbitrary cut-off values selected by the authors in order to stratify groups with better outcomes vs worse outcomes (i.e. cardiovascular events)

^b^Mean values

The selected studies with available data on adverse cardiac outcomes involved 676 participants hospitalized with ADHF (69% male, mean age 68 years). The participants had a high prevalence of comorbidities: 42% had coronary artery disease, 52% belonged to New York Heart Association (NYHA) class III/IV, at least 29% had diabetes mellitus (no data was found in one study), and 56% had hypertension. The median follow-up time was 272 days (range 12–730 days). LS was assessed by TE (Fibroscan) in four studies, by 2D-SWE (Aplio i800 [Canon Medical System] and Supersonic Aixplorer [Hologic]) in 2 studies, by p-SWE (VTQ, Siemens) in one study.

**Fig. 1:**
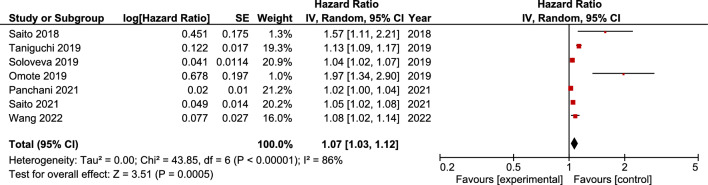
Forest plot assessing the association between liver stiffness and adverse cardiac events

**Fig. 2:**
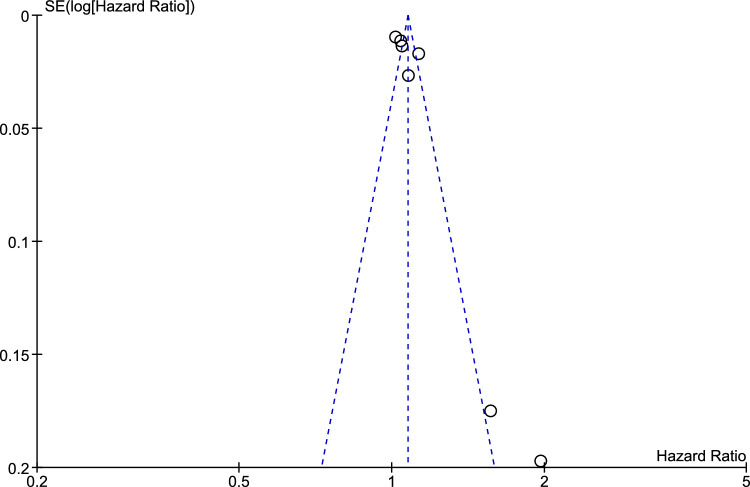
Funnel plot for publication bias

Figure 1 presents the results of pooled meta-analysis showing that increased liver stiffness was associated with higher risk of adverse cardiac events (hazard ratio 1.07 [1.03, 1.12], 95% CI), with substantial heterogeneity [*I*^2^ = 86%]. Figure 2 shows low evidence of publication bias, as indicated by visual inspection of the funnel plot.

## Discussion

In this study, we conducted a systematic review and meta-analysis to summarize the currently available evidence on the diagnostic accuracy of SWE in detecting liver congestion and the prognostic relevance of this non-invasive method in the setting of ADHF.

Despite the remarkable advances in treatment strategies, HF still has high rates of morbidity and mortality, with a major burden to health care systems. In recent years, many efforts have been made to identify prognostic factors to prevent and modify the course of the disease; non-invasive methods to evaluate organ congestion, which is the main driver of cardiac decompensation, have attracted research interest [24, 25].

The heart and the liver are in close relation to each other [26] and the venous congestion has a pivotal role in cardio-hepatic interactions [27]. In fact, systemic venous congestion rises neurohormonal activation, decreases plasma natriuretic peptide, leads to HF progression and multiple organ failure [28]. HF causes increased right-sided filling pressure, leading to LS increase and contributing to worsening liver function; on the other hand, HF is associated not only to hepatic congestion (right-sided HF), but also to reduced arterial flow to the liver, configuring “hypoxic hepatopathy” (left-sided HF) [29].

Conclusively, HF “per se” may lead to irreversible liver disease, and high LS values can result from congestion and liver disease (provided that concurrent etiologies of liver diseases are excluded) [8].

In recent years, several researchers have explored the prognostic value of LS in patients with ADHF and without a primary liver disease.

The present meta-analysis, including seven observational, single-centre, prospective studies involving 676 adult individuals hospitalized for ADHF from different geographical locations, shows that LS is an independent, prognostic marker of cardiovascular outcomes in patients with HF without other known primary liver diseases.

Median LS values were abnormal at baseline and improved at discharge, even though patients with primary significant liver disease or acute hepatitis were excluded and the obvious explanation of this finding is that passive liver congestion in the setting of HF impairs shear wave distribution and leads to increase of LS, which is affected by effective medical treatment.

Patients with a history of alcohol abuse demonstrated higher values of LS on both admission and discharge, but they had no difference in LS change, indicating that LS measurements during hospitalization could apply to a broader spectrum of HF patients, including patients with ongoing toxic liver exposure [25]. Anyhow, only two of the included studies performed a variables adjustment for in the primary outcomes.

The results of the present study expand those coming from a previous meta-analysis, which focused on the association of LS and/or serum biomarkers with cardiovascular outcomes in patients with HF. This paper, by Khan et al., included three studies with available data on adverse cardiac outcomes comprising 792 participants hospitalized with ADHF (37% female, mean age 71 years); the LS was assessed by TE in only two studies included in our analysis [30, 31] and by serum laboratory testing (LFSs—non-alcoholic fatty liver disease fibrosis score) in the third study [32]. The pooled analysis showed that increased LS was associated with higher risk of adverse cardiac events (hazard ratio 1.15, 95% CI 1.04–1.28, *p* = 0.006, *I*^2^ = 60%) [2].

Interestingly, LS seems to be associated with the severity of HF, revealing a part of the complex mechanisms between left- and right-side dysfunction. In fact, Taniguchi et al. showed that higher LS values are associated with advanced NYHA functional class and, together with lower hemoglobin, hematocrit, and sodium levels suggesting increased volume status, with elevated left-sided filling pressure (higher E/e’ and larger left atrial diameter). Conclusively, right-sided filling pressure can be affected by left-sided filling pressure and volume status, together contributing to the severity of HF [30].

Concordant with these findings, et al. reported the associations between parameters of liver congestion (right-sided HF) and liver hypoperfusion (left-sided HF) determined by abdominal ultrasonography and liver function tests, right-heart catheterization and echocardiography, as well as its prognostic impact in HF. Decreased cardiac output was associated with celiac hypoperfusion, and low peak systolic velocity was associated with impaired right and left systolic function assessed by echocardiography [28].

Although all the studies included in our meta-analysis found a positive correlation between an increased LS and a poor prognosis, we did not try to find a “common” LS cut-off value because a significant inter-system variability in LS measurements precludes direct comparison of results obtained with equipment of different manufacturers. In fact, most of the studies included in this metanalysis reported values from 8.0 to 14 kPa [18, 25, 32], and a mortality rate of 20.8% in patients with LS above 8.0 kPa [33] in the HF scenarios was reported. Using a pSWE technique in patients with HF, with a multivariate analysis it was demonstrated that the changes in LS significantly correlated with changes in CVP, and that a LS cut-off value of 7 kPa could predict a CVP > 10 mmHg with 89.6% sensitivity and 87.5% specificity [34, 35].

To summarize, most of studies suggest that patients with acute HF usually have LS values above 7.65 kPa, which often correlate with NT-proBNP levels [36] supporting its strict connection with congestion.

In our meta-analysis we did not try to establish a specific cut-off value by merging data of different elastography devices as this was deemed methodologically inappropriate; on the other hand, the use of hazard ratios and corresponding standard errors does not incur in this kind of error.

In this light, LS measurement in HF patients may become even more useful in monitoring changes over time, as every patient become his/her own control in subsequent measurements (at admission and after decongestant therapy).

## Conclusions

HF population represents a high-risk patient group due to high rates of morbidity and mortality; the early identification of patients with high risk of worse prognosis remains the greatest challenge.

In the ADHF LS reflects congestion secondary to volume and pressure overload and/or inadequate liver perfusion due to reduced cardiac output, and it may be especially helpful to rapidly assess hemodynamic status with a non-invasive technique.

The studies included in our meta-analysis demonstrate a prognostic value of LS in this setting of patients; a possible future goal would be to determine a specific vendor-neutral cut-off value that helps to discriminate between patients at low or high risk for poor cardiovascular outcome (similar to the vendor-neutral “rule of 4” suggested by the Society of Radiologists in Ultrasound [SRU] to classify patients with or without compensated advanced chronic liver disease) [37].

In conclusion, further studies are warranted to evaluate whether the adoption of LS in risk prediction models for patients with HF may improve the prognostic accuracy as compared to currently available algorithms.

## Data Availability

All data from the meta-analysis have been taken from the included study and therefore available to all readers.
